# Resonant Magnetic Field Sensors Based On MEMS Technology

**DOI:** 10.3390/s91007785

**Published:** 2009-09-30

**Authors:** Agustín L. Herrera-May, Luz A. Aguilera-Cortés, Pedro J. García-Ramírez, Elías Manjarrez

**Affiliations:** 1 Centro de Investigación en Micro y Nanotecnología, Universidad Veracruzana / Calzada Ruiz Cortínes 455, 94292, Boca del Río, Veracruz, Mexico; E-Mail: jagarcia@uv.mx; 2 Depto. Ingeniería Mecánica, Campus Irapuato-Salamanca, Universidad de Guanajuato / Carretera Salamanca-Valle de Santiago km 3.5+1.8 km, Salamanca, Guanajuato, Mexico; E-Mail: aguilera@salamanca.ugto.mx; 3 Instituto de Fisiología, Benemérita Universidad Autónoma de Puebla / 14 sur 6301, CU, San Manuel, 72750, Puebla, Puebla, Mexico; E-Mail: emanjar@siu.buap.mx

**Keywords:** Lorentz force, magnetic field sensors, Microelectromechanical Systems (MEMS), resonant structures

## Abstract

Microelectromechanical systems (MEMS) technology allows the integration of magnetic field sensors with electronic components, which presents important advantages such as small size, light weight, minimum power consumption, low cost, better sensitivity and high resolution. We present a discussion and review of resonant magnetic field sensors based on MEMS technology. In practice, these sensors exploit the Lorentz force in order to detect external magnetic fields through the displacement of resonant structures, which are measured with optical, capacitive, and piezoresistive sensing techniques. From these, the optical sensing presents immunity to electromagnetic interference (EMI) and reduces the read-out electronic complexity. Moreover, piezoresistive sensing requires an easy fabrication process as well as a standard packaging. A description of the operation mechanisms, advantages and drawbacks of each sensor is considered. MEMS magnetic field sensors are a potential alternative for numerous applications, including the automotive industry, military, medical, telecommunications, oceanographic, spatial, and environment science. In addition, future markets will need the development of several sensors on a single chip for measuring different parameters such as the magnetic field, pressure, temperature and acceleration.

## 1. Introduction

Microelectromechanical Systems (MEMS) technology includes products such as automobile airbag systems, display systems and inkjet cartridges. It not only reduces the device dimensions on the order of micrometers, but also integrates the mechanical and electronic components on a single chip. This technology allows the design of portable devices such as gyroscopes, accelerometers, micromirrors, and pressure sensors [1-4]. Automotive industry is one of the principal markets for MEMS technology, although this market has been affected due to the global economic downturn in 2008 and 2009. This limits the growth of MEMS market, but it has bright prospects in consumer electronic products and the cell phone industry [5]. For example, new cell phones will include several MEMS devices such as gyroscopes, accelerometers and magnetic field sensors for their Global Positioning System (GPS) and MEMS technology is expected to be a key innovation driver in the cell phone industry. New features are expected to account for 60% of the total MEMS market by 2012. The worldwide MEMS market reached $48 billion dollars in 2005, $72 billion in 2007 and is expected to reach $95 billion by 2010 [5].

Magnetic field sensors have a great potential for numerous applications such as magnetic storage, automotive sensors, navigation systems, non-destructive material testing, security systems, structural stability, medical sensors, and military instruments [6]. Among the different kinds of magnetic field sensors, the Superconducting Quantum Interference Device (SQUID) is the most sensitive sensor, achieving a magnetic field resolution (minimum detectable magnetic field) on the order of several fT [7]. This sensor has a power consumption of several watts [6] and its operation is based on two effects: flux quantization and Josephson effects (observable only in the presence of superconductivity) [7]. The device is used mainly in neuromagnetism (with signal levels of pT or lower), magnetic resonance and geology applications [8]. Unfortunately, the commercial success of SQUID-based applications is still limited due to its high price that, in most cases, overrides its advantages with respect to other magnetic sensors. In addition, SQUID sensors operate at low temperatures and have high sensitivity to electromagnetic interference, requiring a sophisticated infrastructure (liquid helium supply, glass-fiber-reinforced epoxy Dewar vessels, and electromagnetic shielding) that restricts their applications.

On the other hand, magnetic sensors using the Hall effect as their principle of transduction are commonly fabricated on standard Complementary Metal-Oxide Semiconductor (CMOS) technology. Hall effect sensors have low cost and are used for measuring linear position, angular position, velocity and rotational speed. In general, they are applicable for a sensitivity range of 1 to 100 mT and have a power consumption between 100 and 200 mW, an offset of several mT, and a die size less than one millimeter. These sensors can measure either constant or varying magnetic field: the upper frequency limit is about 1 MHz and operate well in the temperature range from −100 to +100 °C [9]. However, magnetic sensors based on silicon may have intrinsic limits to their sensitivity and resolution, which may limit future performance gains [10]. In addition, they need temperature compensation circuits that can include temperature sensor and operational amplifiers (op-amps).

Search coil sensors only detect time-varying magnetic fields based on Faraday's law of induction. Commonly, they can measure magnetic fields above 20 fT [11], and use a ferromagnetic core with high permeability inside of a coil in order to increase their sensitivity. Typical sizes of the coil are between 0.05 to 1.3 m and it has a power consumption ranging from 1 to 10 mW. These sensors have a useful frequency range from 1 Hz to 1 MHz, and are found on roads for traffic control [12]. The miniaturization of these sensors decreases their sensitivity and they cannot detect static magnetic fields.

Fluxgate sensors measure the static or low frequency magnetic field and are sensitive to both the field direction and field magnitude from 10^−2^ to 10^7^ nT with a resolution of 100 pT [9]. Fluxgates are the most widely used sensors for compass navigation systems, but they are also used for detecting submarines, geophysical prospecting, airborne magnetic field mapping, and measurement of electrical current [13,14]. However, these sensors have a complex fabrication of the magnetic core and the coils [15], as well as high mass and power consumption. Any reduction of their mass and power decreases both their sensitivity and stability [16]. The frequency response of the sensor is limited by the excitation field and the response time of the ferromagnetic material. They can be operated up to 100 kHz and have a size on the order of several millimeters, and a power consumption around 100 mW. In order to miniaturize fluxgate sensors two problems have to be solved: the miniaturization of the coils and the integration of the magnetic core [17].

Anisotropic Magnetoresistive (AMR) sensors can achieve a sensitivity range from 10^−1^ to 10^7^ nT [6,18], a typical resolution of 10 nT, and have a size of a few millimeters [19]. These sensors are based on the anisotropic magnetoresistive effect that occurs in ferromagnetic transition metals, in which their electrical resistance depends on the angle between the electrical current and the direction of magnetization. Therefore, an external magnetic field affects the direction of the magnetization, causing a variation of the electrical resistance. The AMR sensors have low sensitivity to mechanical stress and a power consumption of few milliwats [19]. Their applications include traffic counting, earth field sensing, electronic compasses, navigation systems, and wheel speed sensors for Anti-Block System (ABS). These sensors are saturated at small magnetic fields (about several mT) and need a complex resetting procedure; in addition, their sensitivity is degraded when the power consumption is decreased [20]. They can be operated over an extremely wide temperature range (above +200 °C), but the standard sensor packages are limited for temperatures below +150 °C.

Giant magnetoresistive (GMR) sensors have a large shift in the electrical resistance when their thin layers (a few nanometers) of ferromagnetic and non-magnetic materials are exposed to a magnetic field [21]. Generally, they detect magnetic fields from 10 to 10^8^ nT [6] and have a die size close to 1 mm. GMR sensors operate at temperatures above +225 °C, although, they have higher both offset and sensitivity temperature dependence than AMR sensors [22]. Unfortunately, a large magnetic field (close to 1 T) may irreparably damage GMR sensors. These sensors have found many applications, including magnetic read heads [23], vehicle detection and car speed monitoring [24], pneumatic cylinder position sensing, crankshaft position sensors, current detection, and noiseless locking mechanisms [25].

Fiber optic sensors exploit the magnetostrictive effect for measuring magnetic fields, whereby the dimensions of the magnetostrictive material change when it is placed inside an external magnetic field. This material is bonded over a piece of optical fiber, which is used as a leg of a Mach-Zender interferometer that measures the strain of the fiber when is exposed to a magnetic field. These sensors have a sensitivity range from 10^−2^ to 10^6^ nT and are immune to electromagnetic interference (EMI) [6,26]. A problem of this sensor is the identification and incorporation of high magnetostrictive materials into a fiber by appropriate bonding or coating [27]. In addition, both temperature and pressure shifts affect the operation of this sensor.

Recently, MEMS technology has been seriously considered as a candidate for the development of sensors due to the fact that they present several advantages such as small size, light weight, low-power consumption, minimum cost, high functionality, and better sensitivity and resolution. This technology can integrate mechanical and electronic components on a common substrate, achieving the realization of complete systems-on-a-chip. Thus, new resonant magnetic field sensors based on MEMS technology have been developed by some research groups, which show important advantages on their performance. For instance, these sensors are compact and smaller (on the order of micrometers) than several conventional technologies (e.g., fluxgate sensors, SQUIDs, optical fiber sensors, and search coil sensors); therefore, they can be placed closer to low-magnetic field sources for increasing their output signal. In addition, they may be lighter, faster and cheaper than their macroscopic counterparts. These sensors use resonant structures that exploit the Lorentz force principle for detecting magnetic fields. Generally, they measure the displacement of resonant structures exposed to external magnetic fields through capacitive, piezoresistive and optical sensing techniques.

This paper presents a review of different resonant magnetic field sensors based on MEMS technology, describing their operation principles, advantages and drawbacks, some applications, trends and challenges. From these sensors, the optical and piezoresistive techniques allow simple structural configurations that need a minimum of read-out electronics circuitry.

Following the introduction, the paper is organized as follows. Section 2 presents the operation principle of resonant structures and the parameters that affect their resonant frequencies. In addition, this section describes the principal advantages and drawbacks of several resonant magnetic fields sensors based on MEMS technology, which use piezoresistive, optical, and capacitive sensing techniques. Section 3 discusses the potential applications of magnetic field sensors in sectors such as automotive, military, medical, oceanographic, spatial, and environment science. Section 4 presents some trends and challenges of the magnetic field sensors, and finally the paper ends with the conclusion and an outline of further work.

## 2. Resonant Magnetic Field Sensors

In this section, the operation principle and detection techniques of MEMS magnetic field sensors based on resonant structures are presented. Most resonant magnetic field sensors exploit the Lorentz force principle, in where a Lorentz force increases the displacement of a resonant structure that can be measured with optical, piezoresistive, and capacitive sensing techniques.

### 2.1. Operation Principle of Resonant Structures

A resonant structure presents an amplified response to an excitation source applied with a frequency equal to the resonant frequency(ies) of the structure. This amplification is caused by the efficient transfer of the energy from the excitation source to the structure [28]. A structure generally has an infinite number of resonances or vibration modes; thus, a resonant sensor uses a structure that operates at one of these frequencies (generally the first vibration mode or frequency is the most used). Therefore, a sensor based on a resonant structure can achieve larger output signals, increasing its sensitivity.

**Figure 1.**
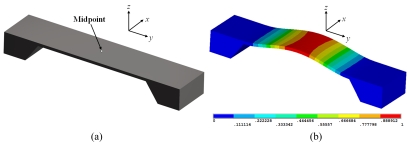
(a) Clamped-clamped beam and (b) its associated first vibration mode.

Resonant magnetic field sensors use structures that are excited at their resonant frequencies by electrostatic forces or Lorentz forces. These structures commonly are integrated by clamped-free/clamped-clamped beams, torsion/flexion plates or an array of them. The application of external magnetic fields alters the deflections of the resonant structure, which can be detected through optical, capacitive, and piezoresistive sensing techniques. For instance, a resonant structure based on a clamped-clamped beam has its first resonant frequency associated to its first flexural vibration mode, as shown in Figure 1(a,b).

**Figure 2.**
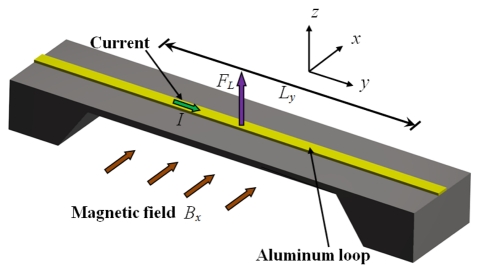
Schematic view of the Lorentz force principle acting on a clamped-clamped beam.

If this beam is exposed to an excitation source with a frequency equal to its first resonant frequency, then the beam will have a maximum deflection at its midpoint. The excitation source can be a Lorentz force due to the interaction between an external magnetic field and an ac excitation current. For this, an aluminum loop is placed on the clamped-clamped beam surface and an excitation current (*I*) flows inside it with a frequency equal to the first beam-resonant frequency (see Figure 2). When the beam is exposed to an external magnetic field (*B_x_*) in the *x*-direction, then a Lorentz force (*F_L_*) is generated. This force can be determined by:

(1) $$F_{L}=IB_{x}L_{y}$$

where *L_y_* is the length of aluminum loop perpendicular to the magnetic field.

The Lorentz force acts as an excitation source on the clamped-clamped beam, causing an amplified deflection on the midpoint. Thus, the magnitude of the beam deflection depends on the Lorentz force amplitude, which is directly proportional to *I* and *B_x_*.

The Rayleigh method is an efficient alternative for determining the first resonant frequency of continuous structures. This method finds the resonant frequency (*f_res_*) through the ratio of the maximum potential energy (*U_max_*) to the maximum kinetic energy (*T_max_*) of the structure [29]. Therefore, the *f_res_* is obtained by:

(2) $$f_{\text{res}}=\frac{1}{2\pi }{\left(\frac{U_{\max}}{T_{\max}}\right)}^{1/2}$$

with:

(3) $$U_{\max}=\int_{L}EI{\left(\frac{\partial^{2}z(y)}{\partial y^{2}}\right)}^{2}dy$$

(4) $$T_{\max}=\int_{L}\rho A{\left(z(y)\right)}^{2}dy$$

where *L* is the length of the resonant structure, *E* is the elasticity modulus, *I* is the cross-sectional moment of area, *ρ* is the density, *A* is the cross-sectional area, and *z*(*y*) is the function of the deflection of the resonant structure.

The first resonant frequency of a structure is directly proportional to its elasticity modulus and cross-sectional moment of area, and inversely proportional to its density and cross-sectional area. In addition, residual stresses on the structure can affect its resonant frequency [30]. Therefore, a variation in these parameters will change the magnitude of the resonant frequency.

Another important parameter in the performance of resonant structures is the damping effect, which limits the maximum amplitude of the structure [31]. The damping level of a resonant structure is determined using its quality factor *Q*, which measures the amount of losses during operation of the resonant structure. *Q* is defined as the ratio of the total energy stored in the structure (*E_M_*) to the energy factor lost per cycle (*E_C_*) due to the damping effect:

(5) $$Q=2\pi \frac{E_{M}}{E_{C}}$$

**Figure 3.**
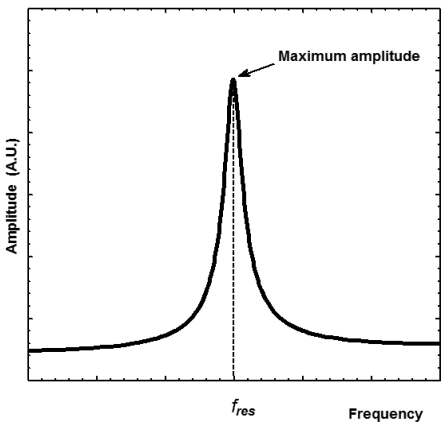
Typical amplitude (arbitrary units) response of a resonant structure with a high quality factor.

A high quality factor implicates a pronounced resonance of the structure (see Figure 3), which can improve the performance and resolution of the resonator. If the structure has a high quality factor then little energy will be needed to keep the resonance at constant amplitude and the electronic circuitry will have a minimum effect on the resonant frequency [31]. Also, a high quality factor indicates that the resonant structure has low sensitivity to mechanical disturbances from its surroundings (e.g., mechanical vibrations) [32].

Generally, total quality factor (*Q_T_*) of a resonant structure depends on the following three damping sources [32]: 1) the energy dissipated to a surrounding fluid (1/*Q_f_*), 2) the energy coupled through the structure's support to a surrounding solid (1/*Q_l_*), and 3) the energy lost internally within the structure's material (1/*Q_i_*). Then, *Q_T_* is given by:

(6) $$\frac{1}{Q_{T}}=\frac{1}{Q_{a}}+\frac{1}{Q_{l}}+\frac{1}{Q_{i}}$$

At atmospheric pressure, commonly the largest energy lost of a resonant structure is due to energy dissipated to a surrounding fluid. This energy loss is due to the interactions of the resonant structure with the surrounding fluid (e.g., air), and its value depends on the surrounding fluid pressure, the nature of the fluid, size and shape of the resonant structure, the type of vibration, and its distance (gap) respect to adjacent surfaces [32]. The quality factor (*Q_a_*) associated to this energy loss has an important increment when the fluid pressure decreases to values close to vacuum [33,34]. In this case, the resonant structure will have larger amplitudes that increase the sensitivity and resolution of a resonant sensor. Hence, this sensor will improve its performance using a vacuum package.

The energy dissipated by the structural damping (1/*Q_l_*) is due to the energy coupled from the resonant structure by means of its supports to the surrounding structure. This energy can be reduced through an adequate design of the structure such as a balanced resonant structure, locating the structure's supports at the nodes of its vibration mode, and using a decoupling system between the structure and its support [32].

Finally, the *Q_i_* is related with the energy loss mechanisms inside the material of the resonant structure. These internal energy losses are due to the thermoelastic damping, phonon interaction, and the movement of dislocations and scattering by impurities [32,35,36].

Most of the resonant structures based on MEMS technology are fabricated on silicon wafers, which have an elastic modulus dependent on the temperature. For example, the silicon elastic modules as a function of the temperature for 250 K ≤ *T* ≤ 600 K is calculated by [37,38]:

(7) $$E\left(T\right)=E_{0}\left(1-9.4x10^{-5}T\right)$$

where *T* is the temperature in Kelvin (K) and *E_0_* is the elastic modulus of silicon at room temperature.

In addition, the thermal expansion coefficient of silicon (*α_Si_*) increases with temperature and its magnitude for 120 K ≤ *T* ≤ 1500 K is determined by [38]:

(8) $$\alpha_{\text{Si}}(T)=3.725x10^{-6}\left(1-e^{-5.88x10^{-3}\left(T-124\right)}\right)+5.548x10^{-10}T$$

The thermal conductivity of silicon (*k_Si_*) increases with temperature, according to the following expression for 300 K ≤ *T* ≤ 400 K [38]:

(9) $$k_{\text{Si}}(T)=309-0.51T$$

The temperature influences on the performance of a resonant sensor, where an increment of the temperature affects the material properties (e.g. the elastic modulus, thermal expansion coefficient, and thermal conductivity) and originates stresses inside of the resonant structure [38]. A variation in the elastic modulus alters the resonant frequency of the sensor and the generation of excessive internal stresses causes strains in its structure, changing the microstructure of its material [39]. These internal stresses shift the resonant frequency of the structures; for instance, in the case of clamped-clamped and clamped-free beams, compressive internal stresses reduce the resonant frequency of the beams. However, they have an increment in their resonant frequency when are exposed to tensile internal stresses [30]. Also, high temperatures may produce corrosion, wear-out, and performance degradation of a resonant sensor [40]. Considering these weaknesses on the MEMS resonant sensors, the temperature stability is a critical issue during the operation of the resonant sensor and it must be controlled through compensation electronic circuits.

The surrounding fluid pressure on the resonant structure is another parameter that affects its resonant frequency. For instance, an excessive pressure on clamped-clamped and clamped-free beams can induce tensile or compressive stresses, altering their resonant frequencies.

### 2.2. Piezoresistive Sensing

In this section, resonant magnetic field sensors based on MEMS technology with piezoresistive sensing are discussed.

**Figure 4.**
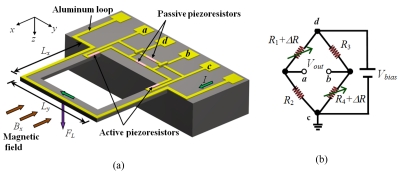
(a) Schematic view of a resonant magnetic field sensor based on two U-shaped clamped-free microbeams and (b) its associated Wheatstone bridge.

Figure 4 shows a depiction of the operating principle of a MEMS magnetic field sensor that uses a resonant structure. The structure is made up by two U-shaped clamped-free microbeams, which are connected to a silicon substrate. The sensor uses a piezoresistive sensing through a Wheatstone bridge with two active (placed on the microbeams) and two passive piezoresistors (deposited on the substrate). The active piezoresistors can shift their resistance magnitudes, whereas, the passive piezoresistors have fixed-value resistances. Using an aluminum loop, an alternating excitation current (*I*) is supplied with a frequency equal to the resonant frequency of the microbeams. When the structure is exposed to an external magnetic field (units teslas) *B_x_* in the *x*-direction, then a Lorentz force is generated [Equation (1)].

The Lorentz force originates a longitudinal strain (*ε_x_*) on the two active piezoresistors changing their resistance values given by:

(10) $$\Delta R=G{ɛ}_{x}R$$

where Δ*R* is the resistance variation of each variable piezoresistor, *G* is the gauge factor of the piezoresistors, and *R* is the resistance of each piezoresistor.

The change in the resistance of the active piezoresistors produces an output voltage shift (*V_out_*) of the Wheatstone bridge. For this case, *V_out_* is determined by:

(11) $$V_{\text{out}}=\frac{\Delta R}{2R+\Delta R}V_{\text{bias}}$$

where *V*_bias_ is the bias voltage of the Wheatstone bridge.

The sensor sensitivity (*S*) is obtained as the ratio of the variation of the output voltage to the range of external magnetic field (Δ*B_x_*):

(12) $$S=\frac{\Delta V_{\text{out}}}{\Delta B_{x}}$$

Consequently, the magnitude of the external magnetic field is measured through the output voltage of the Wheatstone bridge. Piezoresistive sensing is simple and easy to use in resonant magnetic field sensors based on MEMS technology. In the next paragraphs, three kinds of these sensors with piezoresistive sensing are presented.

**Figure 5.**
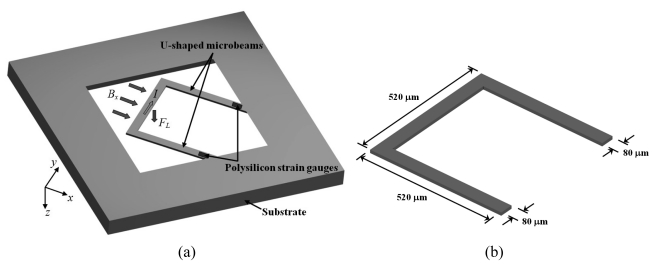
(a) Schematic view of operation principle and (b) dimensions of the resonant U-shaped microbeams used by the magnetic field sensor designed by Bernoulle *et al.* [41].

Beroulle *et al.* [41] developed a magnetic field sensor with resonant U-shaped microbeams of silicon with 80 μm width and 520 μm length [see Figure 5(a)]. The microbeams contain a planar aluminum coil of 80 turns (not represented) and two piezoresistive strain gauges of polysilicon connected to a Wheatstone bridge. A Lorentz force (*F_L_*) is obtained from the interaction between a magnetic field (*B_x_*) and an electrical current (*I*) flowing through the coil, which deflects the microbeams and strains the piezoresistive gauges. Thus, the output voltage of a Wheatstone bridge shifts as a function of the magnetic field applied. The dimensions of the U-shaped microbeams are showed in Figure 5 (b).

Beroulle's sensor is fabricated using an industrial CMOS process, followed by a post-process to release its microbeams. The sensor has a resonant frequency of 8.97 kHz, a quality factor of 59, a mass of 750 ng, a sensitivity of 530 mVT^−1^, a theoretical resolution of 10 μT, and a thermal noise of 5.3 nVHz^−1/2^.

**Figure 6.**
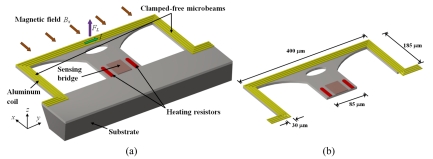
(a) Schematic view of the operation principle and (b) estimate dimensions of the resonant magnetic field sensor developed by Sunier *et al.* [42].

Figures 6(a-b) show a magnetic field sensor designed by Sunier *et al.* [42], which consists of a silicon resonator based on clamped-free microbeams with a planar aluminum coil. This coil has eight turns (four turns with each of the two metal layers) that produces a better uniform distribution of the electrical potential around the piezoresistive detection elements. This sensor exploits the Lorentz force principle and provides a frequency output. The microbeams vibrate steadily at their resonant frequency due to the thermal actuation of two heating resistors. This frequency is a function of the microbeams equivalent mass and spring constant. The interaction between a magnetic field (*B_x_*) and an electrical current (*I*) originates a Lorentz force (*F_L_*) on the tip of the microbeams that changes their equivalent spring (*F_L_* acts like an additional spring force). Thus, the variation of the resonant frequency (Δ*f_res_*) depends on the applied magnetic field *B_x_* as is indicated by:

(13) $$\Delta f_{\text{res}}=\frac{1}{2\pi }{\left(\frac{k-F_{L}/x}{m}\right)}^{1/2}$$

where *k* is the equivalent spring constant of the microbeams, *m* is the effective microbeams mass and *x* is the resonator position (deflection).

The deflections of the microbeams are measured with piezoresistive sensing by means of P-channel Metal Oxide Semiconductor (PMOS) transistors connected in a Wheatstone brigde that is located close to the anchoring end of the microbeams.

The sensor sensitivity (*S*) is obtained as the ratio of the change of the resonant frequency to the range of magnetic field (Δ*B_x_*) applied:

(14) $$S=\frac{\Delta f_{\text{res}}}{\Delta B_{x}}$$

Sunier's sensor is also fabricated with an industrial CMOS process, followed by a post-process of micromachining to release the microbeams. The sensor presents a resonant frequency of 175 kHz, a quality factor of 600, a sensitivity of 60 kHzT^−1^, a resolution of 1 μT, and a power consumption about 5 mW. In addition, this sensor has an efficient continuous offset cancellation technique, a high robustness, low cross sensitivity and competitive cost.

**Figure 7.**
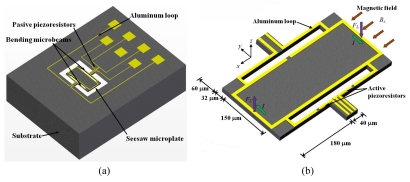
A 3D Schematic view of the (a) structural configuration and (b) operation principle of the resonant magnetic field sensor developed by Herrera-May *et al.* [43].

Herrera-May *et al.* [43] reported a magnetic field sensor based on a silicon resonant microplate (400 × 150 × 15 μm) and four bending microbeams (130 × 12 × 15 μm). This sensor is based on Lorentz force principle and contains an aluminum rectangular loop on the silicon plate, as shown in Figure 7(a). An ac excitation current (*I*) flows on the aluminum loop under the presence of an external magnetic field (*B_x_*), as shown in Figure 7 (b). This interaction originates a Lorentz force (*F_L_*), causing a seesaw motion on the microplate and the bending microbeams. On these are located two active piezoresistors, while on the silicon substrate is located two passive piezoresistors. These four piezoresistors (p-type) are connected in a Wheatstone bridge. The seesaw motion of the bending beams strains the active piezoresistors, which modifies their resistances and the output voltage of the Wheatstone bridge. Then the magnetic field magnitudes are translated into an electrical signal through of the Wheatstone bridge.

This patent pending sensor was designed for Tenaris TAMSA Corporation for measuring residual magnetic fields in welded steel tubes. The fabrication process of the sensor is based on the bulk micromachining protocol of the Microelectronics National Center (CNM) of Spain. This sensor has a resonant frequency of 136.52 kHz, a quality factor of 842, a sensitivity of 0.403 μVμT^−1^, a resolution of 143 nT with a frequency variation of 1 Hz, and power consumption below 10 mW. In addition, the sensor structure is compact and has a high quality factor at ambient pressure. However, the sensor registered an offset and linearity problems at low magnetic fields range.

The resonant sensors with piezoresistive sensing require simple readout circuits and present high sensitivity and low manufacture-cost. Although, they need a careful packaging, trimming, and compensation circuits for reducing the effect of the thermal fluctuations on their performance.

### 2.3. Optical Sensing

In this section, the optical sensing used in resonant magnetic field sensors based on MEMS technology is presented.

**Figure 8.**
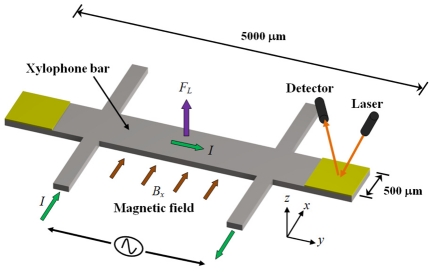
Schematic view of the operation principle of a magnetic field sensor based on a resonant xylophone microbeam designed by Zanetti *et al.* [44].

A xylophone resonator used as magnetic field sensor with optical sensing was reported by Zanetti *et al.* [44]. The xylophone consists of a rectangular microbar supported (at the nodes of its fundamental vibration mode) by four microbeams, as shown in Figure 8. An ac drive current (*I*) is supplied at the resonant frequency of xylophone under an external magnetic field (*B_x_*), producing a Lorentz force that deflects the microbar. This defection is optically sensed through a miniature laser, where it illuminates a xylophone free end and the deflection of the reflected light beam is synchronously detected with a position sensitive detector. Thus, the xylophone deflections are proportional to the applied magnetic fields. A background field is generated by a calibration coil to maintain a proper dynamic range and to inject fields when the xylophone resonance is lost. In addition, the magnitude of the drive current can be adjustable through a feedback loop. The Zannetti's sensor with dimensions of 5000 × 500 × 250 μm has a quality factor around of 7000, a resolution about 1 nT and a power consumption of few milliwatts.

**Figure 9.**
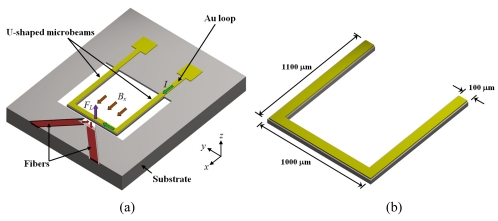
(a) Schematic view of an operation principle and (b) dimensions of a magnetic field sensor with two fibers located in curved channels developed by Keplinger *et al.* [45,46].

**Figure 10.**
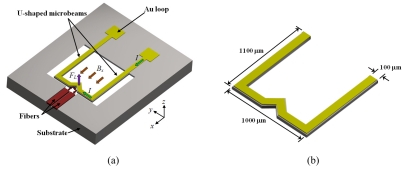
Schematic view of the magnetic field sensor with two parallel fibers and a cranked microbeam designed by Keplinger *et al.* [45,46].

Keplinger *et al.* [45,46] presented a resonant magnetic field sensor using U-shaped silicon microbeams and an optical readout system. The microbeams have a length of 1,100 μm, a width of 100 μm, a thickness of 10 μm, an overall width of 1,000 μm, and a gold loop with a thickness of 0.5 μm. A magnetic field (*B_x_*) and an ac electrical current (*I*) generate a Lorentz force, which bends the microbeams. These deflections are measured with an optical sensing that uses two-fiber arrangement to avoid the problem of the interfering reflected light. Two different designs to connect both fibers in the same side of the chip are used. Figure 9 shows the first design, in which the emitted light beam is reflected only once at the microbeam front side; however, this design needs a great lateral space from the chip and sensor. Figure 10 shows the second design with a cranked microbeam (1,100 × 1,000 × 10 μm) to allow the parallel alignment of the fibers; although, it requires an approximately perfect vertical front side of the microbeams.

The Keplinger sensor is suitable for measuring magnetic fields from 10 mT to 50 T with moderate excitation amplitudes. The sensor has a large dynamic range and is principally used for applications with high magnetic fields. In addition, the sensor can be used in harsh environments under mechanical vibrations and low temperatures. However, this sensor needs high current magnitudes (about 50 mA) to detect small magnetic fields, which increases the temperature and deformation at the silicon microbeam. It can cause a resonant frequency shift of the microbeams and, hence, the sensor will need calibration. Also, the variations of the environment temperature affect the resonant frequency of the microbeam. The sensor has a resonant frequency around 5 kHz, a resolution of 10 mT, and a power consumption of few milliwatts.

Wickenden *et al.* [47] developed a resonant xylophone microbar of polysilicon to measure magnetic fields. This sensor has a similar performance to that reported by Zanneti *et al.* [44], but presents a smaller size (500 × 50 × 2 μm) than Zannetti's one (5,000 × 500 × 250 μm). Wickenden's sensor detects the magnetic fields through the reflection of a laser diode beam (with an incident angle about 5° from vertical incidence) from one of the free ends of the xylophone microbar. The reflected laser beam is collected using a position sensitive detector. Thus, the microbar deflection amplitude is proportional to the applied magnetic field.

Wickenden's sensor has four 4 μm wide support arms and operates to a resonant frequency of 78.15 kHz, a quality factor about 7000, a thermal noise of 100 pTAHz^−1/2^, an ac current of 22 μA and a pressure around of 4.7 Pa. In addition, the sensor can achieve a resolution on the order of nanoteslas. The relation between the output response of the sensor and the applied magnetic field range has a linear behavior up to 150 μT. The performance of the sensor is affected by pressure and temperature fluctuations, e.g., the resonant frequency of the xylophone shifts with the intensity variation of a broad illumination source or the location of the laser.

**Figure 11.**
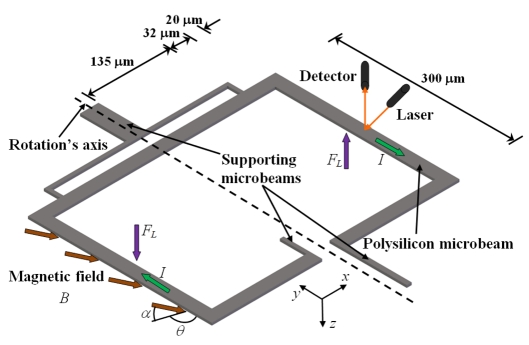
Schematic view of the magnetic field sensor based on resonant polysilicon beams reported by Herrera-May *et al.* [48].

Figure 11(a) shows a magnetic field sensor based on resonant polysilicon microbeams, which is reported by Herrera-May *et al.* [48]. This sensor consists of a microbeams rectangular array with thickness of 1.5 μm and width of 20 μm, as shown in Figure 11(b). An ac current flows (*I*) in the microbeams to their resonant frequency under an external magnetic field (*B*), originating a seesaw motion of the microbeams that can be measured with an optical sensing. Herrera-May *et al.* [48] developed a theoretical model to predict the sensor output response as a function of the magnetic field orientations and ac current magnitudes. Also, they include a theoretical model to determine the influence of the air damping on the quality factor of the sensor. This sensor presents a theoretical sensitivity (as a function of the deflection of the resonant structure) of 530 nmT^−1^, a resonant frequency of 19.4 kHz, a damping ratio of 0.301, an ac excitation current of 574 μA, and a power consumption about 1.2 mW. This sensor can improve its output signal whether the air damping is decreased by means of the pressure reduction (e.g., using a vacuum packaging). Thus, the sensor must operate at low pressures and needs to include an optical detection system.

The resonant magnetic field sensors with optical readout system have immunity to EMI as well as a reduction in their electronic circuitry and weight. However, the optical sensing presents some problems due to the intrinsic losses of the structural imperfections of the sensors and can require complex fabrication processes.

### 2.4. Capacitive Sensing

In this section, some resonant magnetic field sensors based on MEMS technology with capacitive sensing are shown.

**Figure 12.**
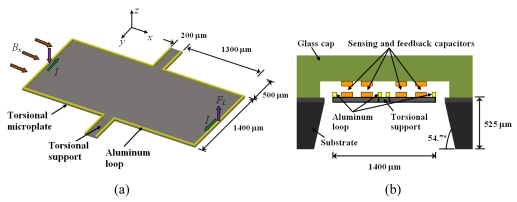
(a) Schematic view of the operation principle and (b) cross-section of the resonant magnetic field sensor developed by Kádár *et al.* [49,50].

Kádár *et al.* [49,50] developed a magnetic field sensor based on a resonant torsional microplate (2,800 × 1,400 × 12 μm) of silicon, which uses a capacitive readout system [see Figure 12(a)]. The sensor contains an aluminum rectangular loop on its surface that active a seesaw motion under an external magnetic field (*B_x_*). The oscillation magnitude is measured through the capacitance variation between the polysilicon electrodes located on the microplate surface and aluminum electrodes placed under a glass packaged [see Figure 12(b)]. Thus, the values of *B_x_* are converted into an electrical domain by means of the capacitor electrodes with 10 μm air-gap. The microplate deflections can achieve large levels if the electrical current in the aluminum loop has a frequency close to the resonant frequency of the microplate.

The Kadar sensor is fabricated using a combination of bipolar processing, micromachining, glass processing and glass-to-silicon anodic bonding at the Delft Institute of Microelectronics and Submicron Technology (DIMES). This sensor with a single loop presents a sensitivity of 500 μVμT^−1^, a resonant frequency about 2.4 kHz, a quality factor of 700, a pressure around 5 Pa, and a power consumption on the order of milliwatts. This sensor requires a complex electronic circuit for the signal processing and can reach a detection limit of 1 nT when it is vacuum-packaged.

**Figure 13.**
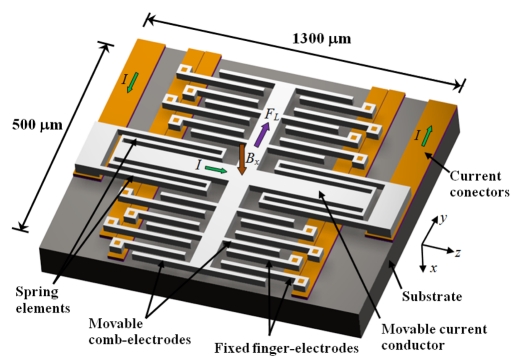
Schematic view (not to scale) of the resonant magnetic field sensor based on movable comb and fixed finger electrodes reported by Emmerich *et al.* [51].

Figure 13 shows a resonant magnetic field sensor reported by Emmerich and Schöfthaler [51], which contains a collection of not only movable comb and fixed finger electrodes, but also a large movable conducting microbeam. The sensor is fabricated using a surface micromachining process and its operation exploits Lorentz force (*F_L_*). This force is generated on the conducting beam when an ac current (*I*) flows through it under the presence of an external magnetic field (*B_x_*). Consequently, the movable electrodes change their distance with respect to fixed electrodes and the capacitance magnitude is altered. Hence, the magnetic field is detected by means of the capacitance variation. The sensor works at resonance and into a vacuum ambient in order to increase its sensitivity.

This sensor has the following results: sensitivity of 820 μVμT^−1^ under an excitation current of 930 μA, a quality factor about 30 at 101 Pa, a resonant frequency of 1.3 kHz, and a resolution close to 200 nT considering a frequency difference of 10 Hz. Furthermore, it presents an offset (approximate 60 μT) due to parasitic couplings of the electronics, unbalanced parasitic capacitances, and a fraction of the residual magnetic field. Also, the sensor requires a complicated fabrication process and a vacuum packaging.

Tucker, Wesoleck and Wickenden [52] designed a resonant magnetic field sensor based on a xylophone microbar that exploits the Lorentz force principle. The xylophone microbar has four support arms located at the nodes of its fundamental vibration mode. The interaction between an ac sinusoidal current to the sensor resonant frequency and an external magnetic field produces a Lorentz force normal to the microbar surface. This force deflects the microbar, changing the capacitance between the movable and fixed electrodes. This capacitance variation is converted to an analog voltage using a modulation-demodulation technique.

This sensor is fabricated using an industrial CMOS process and has a die area around 0.5 mm^2^. The sensor presents a resonant frequency close to 100 kHz, a quality factor about 1,000, a noise of 0.5 nTHz^−1/2^, and a power consumption of 7.5 mW. The sensor needs a complex fabrication process, a vacuum packaging, and a differential switched capacitor modulation and demodulation technique.

**Figure 14.**
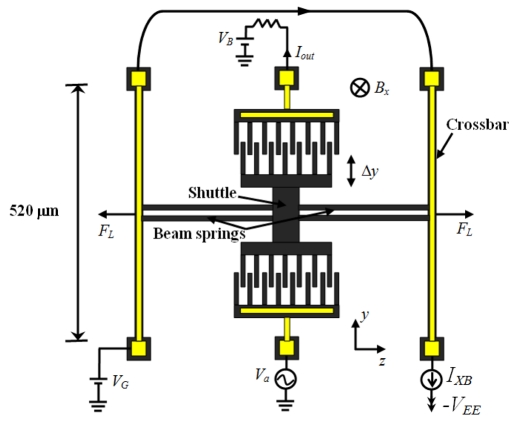
Schematic view of electrostatic resonator used as magnetic field sensor by Bahreyni *et al.* [53].

Figure 14 shows a resonant magnetic field sensor developed by Bahreyni *et al.* [53], which was fabricated in a standard bulk micromachining process without any additional processing steps. This sensor contains an electrostatic resonator (shuttle) connected to two crossbar (520 × 9 × 10 μm) by four microbeam springs (200 × 3 × 10 μm). The resonator is driven and kept into resonance through electrostatic actuation and sensing. A Lorentz force (*F_L_*) normal to the crossbars is obtained with the interaction between a dc current (*I_XB_*) in the crossbars under a magnetic field (*B_x_*) normal to the plane of the sensor. This force produces axial stresses on the microbeams, which change the resonant frequency (*f_res_*) of the shuttle. Then, the signal processing electronics detects the resonant frequency variation which is function of both the magnetic field *B_x_* and current *I_XB_*. In this case, the sensitivity (*S*) of the sensor to *B_x_* is determined by:

(15) $$S=\frac{6\pi I_{\text{XB}}L_{\text{XB}}f_{\text{res}}}{5NLk_{b}}$$

where *L*_XB_ is the length of the crossbars, *k_b_* is the spring constant of each microbeams springs, and *N* and *L* is the number and length of the microbeam springs.

Bahreyni's sensor operates with electrostatic actuation, which presents the following advantages: low-power requirements, ease of manufacture, and simple operation. This sensor has a resonant frequency around 27 kHz, a quality factor of 15,000 at 2 Pa, a mass about 250 ng, a sensitivity of 69.6 HzT^−1^, and a resolution de 217 nT for 10 mA current in crossbars with 2V actuation voltage. However, the sensor needs a vacuum packaging for improving its performance and, in addition the heat produced by the current *I_XB_* changes the resonant frequency of the sensor structure. Also, alterations in the environment temperature affect the sensor performance and its electronic circuits.

The resonant magnetic field sensors with capacitive sensing present a little temperature dependence, but they suffers from parasitic capacitances in the connecting leads that complicate the signal measurements. The parasitic capacitances can be decreased whether electronic circuits are fabricated on the same substrate from the magnetic field sensor. In addition, capacitive sensing requires complicated electronics and vacuum-packaged.

### 2.5. Comparison of Magnetic Field Sensors

The resonant magnetic field sensors based on MEMS technology have simple operation principles, which allow the design of compact and lighter structures integrated by few elements (e.g., clamped-free microbeams, aluminum loop, piezoresistors, and electrodes). They can measure low magnetic fields around nanoteslas; although, the reduction of this level (to magnetic fields on the order of picoteslas) could be achieved with future optimized designs of the resonant structures and electronic circuits. Generally, the sensitivity range of these sensors is adjusted changing the excitation current of the aluminum loop, which help to measure lower or higher magnetic fields. These sensors can be used closer to the magnetic field sources due to their small size and have a power consumption of a few milliwatts. A summary of the main characteristics of these sensors are shown in Table 1.

**Figure 15.**
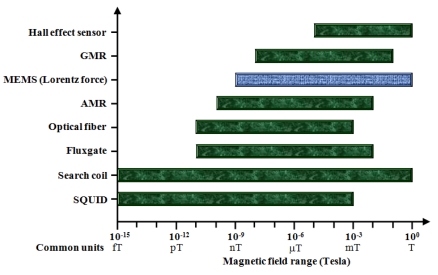
Approximate sensitivity range of different magnetic field sensors [6,16,26].

Figure 15 shows the approximate sensitivity range of the most common magnetic field sensors, including MEMS technology. Also, the characteristics of resolution, noise, power consumption, and minimum size of these sensors are indicated in Table 2. Based on these results, the MEMS sensors could compete with the conventional sensors into numerous applications for measuring magnetic fields higher than 1 nT. The MEMS technology achieves low-cost sensors by means of batch fabrication techniques and their potential integration with integrated circuits (IC) on a same substrate. This is an attractive characteristic of the MEMS sensors for future markets.

## 3. Some Applications

**Figure 16.**
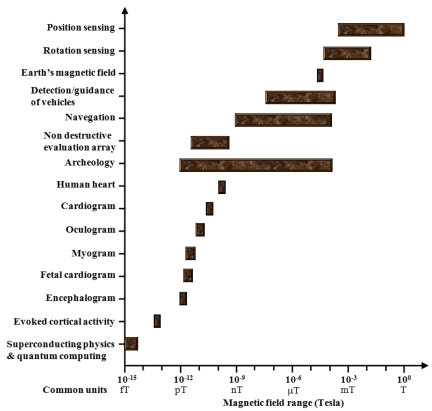
Typical magnetic signals range of several sources and applications [8,13,16,63].

Magnetic field sensors fabricated on MEMS technology offer new capabilities that can be used in industrial sectors such as the automotive, military, medical, oceanographic, spatial, and environment science. A typical magnetic signal range of several sources and applications is shown in Figure 16 [8,13,16,63].

**Figure 17.**
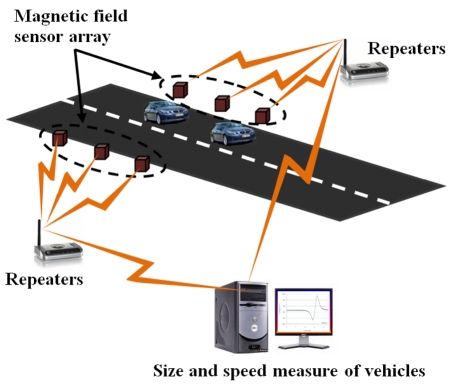
Schematic diagram of the size and speed measure of vehicles.

**Figure 18.**
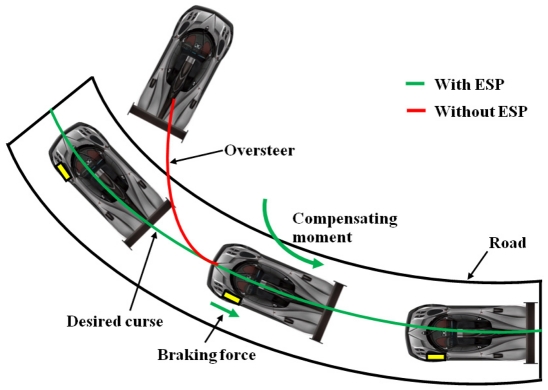
Effect on the vehicle stability achieved with an ESP system.

**Figure 19.**
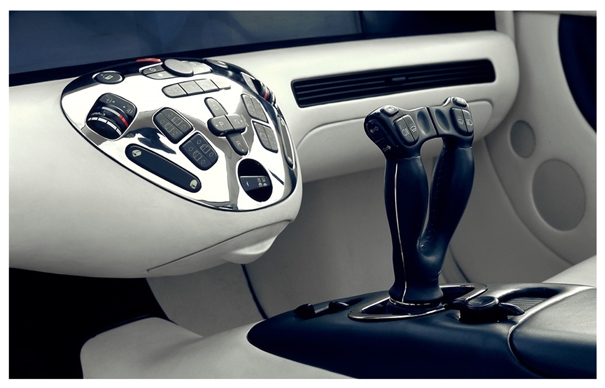
Futuristic Drive-by-Wire system of the Mercedes-Benz Company [69].

In the automotive sector, the magnetic field sensors can be used for on-road vehicle classification through the detection of magnetic disturbances [64]. Figure 17 shows a magnetic field sensor array separated by a small distance, which measure the speed and size of vehicles for traffic classification. The speed control, steering wheel angle, ABS, Electronic Stability Programme (ESP) and recently in the Drive-by-Wire (DbW) system [65,66] are applications of these sensors. ESP system keeps the vehicle dynamically stable in critical situations such as slippery surfaces and hard braking. It needs information about steering-wheel angle, lateral accelerations, yaw rate, and wheel speed that are obtaining using accelerometers, gyroscopes, pressure devices and magnetic field sensors. Figure 18 shows an example of the effect on the vehicle stability achieved with an ESP system. DbW systems replace the conventional mechanical and hydraulic control systems by electronic control systems through electromechanical actuation (that can include magnetic field sensors) and human-machine interfaces for improving the overall automotive safety and performance [67,68]. Figure 19 shows a futuristic DbW system of Mercedes-Benz Company that integrates a sidestick to guide the vehicle, eliminating the brake-pedal [69].

In the military sector, the magnetic field sensors are used to located unexploded ordnance that contains dangerous explosives, propellants or chemical agents [13]. The residual magnetization of ordnance is proportional to its outer volume, shell thickness, length-diameter ratio, relative permeability, and orientation in the geomagnetic field [70]. In addition, the magnetic field sensor can be used to track the autonomous missiles or intelligent ammunition during the flight and to detect submarines [13].

**Figure 20.**
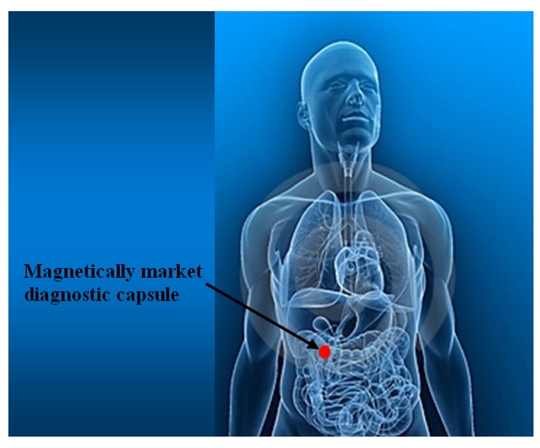
Future medical application of magnetic field sensors for visualizing magnetically market diagnostic capsules.

The medical sector demands magnetic field sensors of high resolution and high sensitivity. For example, the iron overload in patients can be monitored through an array of magnetic field sensors. In addition, studies of brain and heart disorders in patients demand magnetic field sensors with better accuracy and high resolution. Also, the tracking of the location and orientation of instruments in microsurgery are needed. Figure 20 shows a future application to visualize a magnetically market diagnostic capsule in real time inside human body using a magnetic field sensors array (more information in [71]).

In Oceanography, magnetic field sensors can be used for an oceanographic mapping [72] and detection of ships, submarines, mineral deposits, and other magnetic objects [73]. Space Science applications include the measurement of absolute field levels and curl of interplanetary space, and satellite-generated fields [72].

In Environment Science there is a need for portable sensors for geological and archeological prospecting, measurements of magnetic properties of rocks, and detection of magnetic fields on the Earth's surface or atmosphere (e.g., the detection of pipeline corrosion).

## 4. Trends and Challenges

The trend in magnetic field sensors is towards devices with higher sensitivity and resolution, smaller size, lower power consumption and minimum cost, although, the improvement of the sensitivity is not independent of the size, power, and cost, and for each application it is necessary to make a trade-off between these factors [6]. New magnetic field sensors must improve their materials, processing, manufacture, and compatibility with the electronic systems, and need to reduce the cost of the signal processing electronics. In addition, future markets will require the development of multisensors on a single chip for measuring different parameters such as magnetic field, pressure, acceleration and temperature. These multisensors will include several microstructures, transducers, and electronic circuits on a same substrate using monolithic fabrication, which will have important advantages such as compact structural configuration, small size, low cost, minimum power consumption, and high functionality without the degradation of the sensitivity and resolution. This integration is a difficult challenge, in where the MEMS technology can take advantages of conventional technologies.

New applications of magnetic field sensors include the detection of biomolecules [74], which has various advantages over the classical detection method such as rapid results, multi-analyte detection, and low price [74]. Other applications need to evaluate the residual and applied stresses in engineering structures for providing early indications of stress state and eventual failure of the structures [75]. For example, if a magnetic metal is strained then it is transformed from a non-magnetic state to a magnetic state, which is referred as metal magnetic memory (MMM) or the residual magnetic field (RMF) [75]. Another future market is the cell phone industry, which will need electronic compasses based on magnetic field sensors for GPS navigation.

A challenge of the magnetic field sensors is the suppression of any background noise. The performance limit of the future magnetic field sensors will be the variations in Earth's magnetic field due to geological effects. In addition, for the futures magnetic field sensors is recommended: to simplify the basic sensor physics, design rules and functionality; to increase their quality factor and dynamic range; to decrease the size, low power, output response offset and the temperature dependence.

In the future, the resonant magnetic field sensors based on MEMS technology could be used principally in the markets of the automotive industry, communications and consumer electronics products. The cost from these sensors will depend principally from the volume-production quantity and the complex of their packaging and signal processing. For instance, in automotive applications, the AIS326DQ accelerometer from STMicroelectronics Company registered in 2008 a price around $3.75 for a volume production from 100,000 pieces [76]. This accelerometer is manufactured using MEMS technology and it has a capacitive sensing and an IC interface. Thus, the next generation of magnetic field sensors fabricated with MEMS technology can have a low cost for high volume production. Although, first this technology must solve the negative effects on the sensor's operation caused by the temperature and pressure shifts. In addition, it will need to reduce the time in the design phase of the sensor and to optimize its performance. Also, investigations on new materials with better mechanical and electrical properties and the development of reliability tests more efficient on the resonant sensors will be needed.

## 5. Conclusions

A review of magnetic field sensors with resonant structures fabricated on MEMS technology has been presented. This technology has allowed the development of Lorentz-force based magnetic field sensors with specifications such as small size, light weight, low power consumption and high performance. These sensors take advantage the optical, capacitive, or piezoresistive sensing capability in order to detect the magnetic fields variations. The sensors with optical sensing decrease their electronic circuitry and weight; however, they can require a complex fabrication process. Moreover, the sensors with capacitive sensing have little dependence on the temperature, but need vacuum-packaging and complex electronic circuitry. The piezoresistive sensing capability allows simple readout circuits and a straightforward fabrication process steps.

Future applications of magnetic sensing will involve sensors with better resolution, smaller dimensions and lower cost. Thus, new magnetic field sensors, as a whole, must improve their fabrication process in order to increase their performance as well the quality factor, and consequently, decreasing the offset and thermal dependency. In addition, new markets will need the development of a multisensor on a single chip for measuring different parameters, including magnetic field, pressure, temperature, and acceleration. The overcoming of these difficult challenges can be achieved through MEMS technology due to its important characteristics discussed in this work. However, this technology needs to realize investigations on new materials and to optimize the sensors operation for increasing their resolution and life-time.

## Figures and Tables

**Table 1.** Main characteristics of resonant magnetic field sensors based on MEMS technology.

**Sensor proposed by**	**Sensing technique**	**Resolution (nT)**	**Resonant frequency (kHz)**	**Quality factor**	**Size of resonant structure** (μm × μm)

Beroulle *et al.* [41]	Piezoresistive	2 × 10^3^	8.97	59 @ P_atm_	520 × 520
Sunier *et al.* [42]	Piezoresistive	10^3^	175	600 @ P_atm_	∼400 × 185
Herrera-May *et al.* [43]	Piezoresistive	143	136.52	842 @ P_atm_	400 × 150
Zanetti *et al.* [44].	Optical	1	---*	∼7000 @ P_atm_	5,000 × 500
Keplinger *et al.* [45,46]	Optical	10^7^	5	---*	1,100 × 1,000
Wickenden *et al.* [47]	Optical	<10^3^	78.15	700 @ 4.7 Pa	500 × 50
Herrera-May *et al.* [48].	Optical	∼10^7^	19.40	1.66 @ P_atm_	404 × 300
Kádár *et al.* [49,50]	Capacitive	∼1 nT	2.40	700 @ 5 Pa	2,800 × 1,400
Emmerich and Schöfthaler [51]	Capacitive	∼200 nT	1.30	30 @ 101 Pa	1,300 × 500
Tucker, Wesoleck and Wickenden [52]	Capacitive	<10^3^	100	∼1000 @ 1 P_atm_	<1,000 × 1,000
Bahreyni *et al.* [53]	Capacitive	217 nT	∼27	15000 @ 2 Pa	∼520 × 400

* Data not available in the literature.

**Table 2.** Characteristics of some magnetic field sensors.

**Technology**	**Resolution (nT)**	**Noise (nTHz^−1/2^)**	**Power consumption (mW)**	**Minimum size (mm × mm)**

Hall effect [6,54]	∼10^5^	∼4,000	∼150	<1 ^a^
GMR AAL002-02 [55]	5V 10 @ 1 Hz	10 @ 1Hz	∼5	0.44 × 0.34 ^a^
MEMS (Lorentz force) [49,50,52]	∼1	∼0.5 ^b^	<10	<1 ^a^
AMR HMC1022 [56]	5 V 8.5 @ 10 Hz	5 V 48 @ 1 Hz	∼25	∼ 1 ^a^
Optical fiber [6,26,57]	<1	<10	<1,000	∼100 × 25^c^
Fluxgate [58]	60	∼10^−1^	∼100	5 × 2.5
Search coil [6,13]	20 × 10^−6^	30 × 10^−3^	<10	50 × 25
SQUID [59-62]	∼10 × 10^−6^	∼30 × 10^−3^	∼1.8 W	<10× 10 ^d^

a Die size of the sensor.

b Noise of sensor developed by Tucker, Wesoleck and Wickenden [52].

c Typical size of the sensor.

d Approximation of the chip size of SQUID without considering the cooling system.
